# Supplementary material for: Genetic therapy in a mitochondrial disease model suggests a critical role for liver dysfunction in mortality
Source: eLife. 2022 Nov 21;11:e65488. doi: 10.7554/eLife.65488 (PMC9859037; doi:10.7554/eLife.65488)
Supplement: Figure 8—source code 1. [file elife-65488-fig8-code1.zip › 04-12-2020-ADV-eLife-65488/Figure 8 R code.docx]

R version 3.4.4 (2018-03-15) -- "Someone to Lean On"

Copyright (C) 2018 The R Foundation for Statistical Computing

Platform: x86_64-apple-darwin15.6.0 (64-bit)

R is free software and comes with ABSOLUTELY NO WARRANTY.

You are welcome to redistribute it under certain conditions.

Type 'license()' or 'licence()' for distribution details.

Natural language support but running in an English locale

R is a collaborative project with many contributors.

Type 'contributors()' for more information and

'citation()' on how to cite R or R packages in publications.

Type 'demo()' for some demos, 'help()' for on-line help, or

'help.start()' for an HTML browser interface to help.

Type 'q()' to quit R.

> knitr::opts_chunk$set(echo = TRUE)

> if(!require(gee)){install.packages("gee")}

Loading required package: gee

> require(gee)

> lrpprc.data <- read.csv("lrpprcdata.csv")

> lrpprc.data$Genotype<- relevel(lrpprc.data$Genotype, ref = "W")

> my.mdl <- gee(LogTG~Genotype + CRE + Genotype*CRE, id = ExpID, data=lrpprc.data,

+ corstr="exchangeable")

Beginning Cgee S-function, @(#) geeformula.q 4.13 98/01/27

running glm to get initial regression estimate

(Intercept) GenotypeH GenotypeM CRE GenotypeH:CRE GenotypeM:CRE

2.64906724 -0.22759390 0.33732466 -0.43380332 0.39115217 0.04718073

Warning message:

In gee(LogTG ~ Genotype + CRE + Genotype * CRE, id = ExpID, data = lrpprc.data, :

Working correlation estimate not positive definite

> summary(my.mdl)$coefficients

Estimate Naive S.E. Naive z Robust S.E. Robust z

(Intercept) 1.55838340 0.9321202 1.67187 0.6817903 2.2857223

GenotypeH 0.14801804 NaN NaN 0.2328612 0.6356492

GenotypeM 0.71293659 NaN NaN 0.3050740 2.3369300

CRE -0.05819138 NaN NaN 0.1983339 -0.2934010

GenotypeH:CRE 0.01554024 NaN NaN 0.1508394 0.1030251

GenotypeM:CRE -0.61281030 NaN NaN 0.2137285 -2.8672372
