# Supplementary material for: Genetic therapy in a mitochondrial disease model suggests a critical role for liver dysfunction in mortality
Source: eLife. 2022 Nov 21;11:e65488. doi: 10.7554/eLife.65488 (PMC9859037; doi:10.7554/eLife.65488)
Supplement: Supplementary file 1. — (A) List of oligonucleotides used in the study. (B) Prediction of Human LRPPRC binding sites within the human mitochondrial genome (NC_012920.1). The top twenty matches among both strands of the complete mitochondrial genome are shown. The p-values were calculated with the FIMO program and were used for ranking. (C) Prediction of zebrafish Lrpprc binding sites within the zebrafish mitochondrial genome (NC_002333.2). The top twenty matches among both strands of the complete mitochondrial genome are shown. The p-values were calculated with the FIMO program and were used for ranking. [file elife-65488-supp1.docx]

**Supplementary tables**

**Supplementary file 1A:** List of oligonucleotides used in the study

| **Gene Id** | **Molecular assay** | **Forward primer (5’-3’)** | **Reverse primer (5’-3’)** |
| --- | --- | --- | --- |
| *lrpprc ex 22_1* | Genotyping | GATGCTCAAAAGTACATCGCCTTGG | ------ |
| *GBT0235_1* | Genotyping | ------ | GTCAACAAACAGCAACAGAGCTGAAC |
| *mRFP_1* | Genotyping | ------ | ACTCCTTGATGACGTCCTCGGA |
| *lrpprc_2 seq* | Non-invasive enzymatic genotyping | CAAGTCCTCACATTTGTTACC | CTTTCACTGCAGACATGTGG |
| *mRFP_2 seq* | Non-invasive enzymatic genotyping | CGACTACTTGAAGCTGTCCTT | TCGTACTGTTCCACGATGGT |
| *lrpprc* | qPCR | TGATAATGCTGAGGAAGCTCTCAAACTG | CCTTCATCTCCTTCAGTATGTCTAACGC |
| *mt-nd4* | qPCR | ACATCAACTCGGAGCCTGTAAACC | GTGTTGGGATAAGTGTAGCTTCG |
| *mt-cyb* | qPCR | CCCTTACACGATTCTTCGCATTCC | GGTTTCGTGGAGAAATAGCAAGTG |
| *mt-co1* | qPCR | TGGTGCTTGAGCCGGAATAGTAGG | CTCCTGGTTGGCTAAGTTCAGCTC |
| *mt-atp6* | qPCR | CCCTTATCCTCGTTGCCATACTTCTAC | GTTGGTTTGTGAATCGTCCAGTC |
| *mt-atp8* | qPCR | ATGCCTCAGCTTAATCCAAAACCC | GCATCAACTTGAGTTGGGTCATTAGG |
| *eef1a1l1* | qPCR | ACCATCGAGAAGTTCGAGAAGGAAGC | GGTGACGTAGTATTTGCTGGTCTCG |
| *mt-nd1* | Copy number | AGCCTACGCCGTACCAGTATT | GTTTCACGCCATCAGCTACTG |
| *polg* | Copy number | GAGAGCGTCTATAAGGAGTAC | GAGCTCATCAGAAACAGGACT |

**Supplementary file 1B:** Prediction of Human LRPPRC binding sites within the human mitochondrial genome (NC_012920.1). The top twenty matches among both strands of the complete mitochondria genome are shown. The p-values were calculated with the FIMO program and were used for ranking.

|  | **MT gene** | **Genome position** | **Strand** | **p-value** | **Sequence** |
| --- | --- | --- | --- | --- | --- |
| 1 | *MT-CO2* | 8262-8295 | − | 8.73E-05 | GGGCTCTAGAGGGGGTAGAGGGGGTGCTATAGGG |
| 2 | *MT- RNR1* | 1452-1485 | + | 1.09E-04 | TAGTTGAACAGGGCCCTGAAGCGCGTACACACCG |
| 3 | *MT-ND5* | 13958-13991 | + | 1.71E-04 | GCCTTCTTACGAGCCAAAACCTGCCCCTACTCCT |
| 4 | *MT-CO1* | 6941-6974 | + | 2.43E-04 | TCTTTTCACCGTAGGTGGCCTGACTGGCATTGTA |
| 5 | *MT-ATP6* | 8821-8854 | + | 2.48E-04 | TCTATAAACCTAGCCATGGCCATCCCCTTATGAG |
| 6 | *MT-ND3* | 10341-10374 | + | 2.89E-04 | ATCATCATCCTAGCCCTAAGTCTGGCCTATGAGT |
| 7 | *MT-ND4* | 11860-11893 | − | 3.14E-04 | TTCTCCCAGTAGGTTAATAGTGGGGGGTAAGGCG |
| 8 | *MT-CO1* | 6288-6321 | + | 4.66E-04 | TACCCTCCCTTAGCAGGGAACTACTCCCACCCTG |
| 9 | *MT-ND1* | 3676-3709 | + | 4.85E-04 | AACTCAAACTACGCCCTGATCGGCGCACTGCGAG |
| 10 | *MT-ND1* | 3680-3713 | − | 5.71E-04 | ACTGCTCGCAGTGCGCCGATCAGGGCGTAGTTTG |
| 11 | *MT-ND4* | 11956-11989 | − | 5.9E-04 | TATGTAGAGGGAGTATAGGGCTGTGACTAGTATG |
| 12 | *MT-RNR2* | 3000-3033 | + | 6.15E-04 | AGGACATCCCGATGGTGCAGCCGCTATTAAAGGT |
| 13 | *MT-CO3* | 9518-9551 | − | 6.42E-04 | GTGCCCTCCTAATTGGGGGGTAGGGGCTAGGCT |
| 14 | *MT-CO2* | 8159-8192 | − | 6.66E-04 | TTGCTCCACAGATTTCAGAGCATTGACCGTAGT |
| 15 | *MT-TW* | 5531-5564 | − | 7.1E-04 | GCAACTTACTGAGGGCTTTGAAGGCTCTTGGTCT |
| 16 | *MT-ND4* | 11404-11437 | + | 7.1E-04 | ACTCCCTAAAGCCCATGTCGAAGCCCCCATCGCT |
| 17 | *MT-ND3* | 10346-10379 | − | 7.24E-04 | TAGTCACTCATAGGCCAGACTTAGGGCTAGGATG |
| 18 | *MT-CO1* | 6011-6044 | − | 7.24E-04 | TATTCGAGCCGAGCTGGGCCAGCCAGGCAACCTT |
| 19 | *MT-CO1* | 8272-8305 | − | 8.18E-04 | TGCTTCCGTGGAGTGTGGCGAGTCAGCTAAATAC |
| 20 | *MT-CO1* | 2343-2373 | − | 8.36E-04 | TCGAATAAGGAGGCTTAGAGCTGTGCCTAGGACT |

**Supplementary file 1C:** Prediction of zebrafish Lrpprc binding sites within the zebrafish mitochondrial genome (NC_002333.2). The top twenty matches among both strands of the complete mitochondria genome are shown. The p-values were calculated with the FIMO program and were used for ranking.

| **S.no** | **MT gene** | **Genome position** | **Strand** | **p-value** | **Sequence** |
| --- | --- | --- | --- | --- | --- |
| 1 | *mt-co2* | 8122-8152 | + | 2.64E-05 | AAATTCTAATGGCACACCCTGCACAACTAGG |
| 2 | *mt-co1* | 6516-6546 | − | 3.69E-05 | AGTTCAGCTCGGATTAAGAGGCTTAATGCGG |
| 3 | *mt-co1* | 6813-6843 | + | 5.1E-05 | CACCTCTTGCAGGCAACCTTGCCCATGCAGG |
| 4 | *mt-ta/tw* | 6101-6131 | + | 8.2E-05 | GTCTCTGATAGGACTTACAGACGTTACTCCG |
| 5 | *mt-rnr1* | 1047-1077 | + | 1.09E-04 | AGCTTTTACCTAATTTACACATGCAAGTCTC |
| 6 | *mt-nd2* | 5682-5712 | + | 1.35E-04 | AACAACGACCTGACCGAAAAGCCCAATCCTG |
| 7 | *mt-co3* | 10411-10441 | + | 2.3E-04 | ACTTCACATCAGACCACCACTTTGGTTTTGA |
| 8 | *mt-atp6* | 9177-9207 | + | 2.6E-04 | ACTTATCACCGTCCAAACATGATTGACTGGA |
| 9 | *mt-co1* | 7698-7728 | + | 3.55E-04 | TCTTCCCACAACATTTCCTAGGTTTAGCAGG |
| 10 | *mt-nd4* | 11490-11520 | + | 5.32E-04 | ACCTCTTCTAGTACTAACCTGTTGACTTCTT |
| 11 | *mt-nd3* | 10705-10735 | + | 6.97E-04 | AATGCGGATTTGATCCACTAGGGTCCGCCCG |
| 12 | *mt-co2* | 8752-8782 | − | 7.31E-04 | AATTCAAGTGGGACGGCTTCGACTACAATGG |
| 13 | *mt-tp* | 16538-16568 | + | 7.57E-04 | AGATTTTAACTCACACCCCTGACTCCCAAAG |
| 14 | *mt-nd2* | 5690-5720 | − | 9.37E-04 | ATTGCTGCCAGGATTGGGCTTTTCGGTCAGG |
| 15 | *mt-rnr1* | 1682-1712 | − | 9.5E-04 | CACCTCGACCTGACTTTCTGGGCTATGCCCA |
| 16 | *mt-nd4* | 12310-12340 | + | 9.5E-04 | ACTCATAGCCGGACCATAATCCTCGCTCGAG |
| 17 | *mt-rnr2* | 2373-2403 | − | 1.05E-03 | ACTTCTACCCGGAGCTCTTCCCACTCTATTG |
| 18 | *mt-rnr2* | 2324-2354 | + | 1.07E-03 | AGCTACCCCGAGACAGCCTATTTAACTTAGG |
| 19 | *mt-nd1* | 4738-4768 | + | 1.07E-03 | CCTACCAATCGCACTAGCTGGTCTACCCCCA |
| 20 | *mt-rnr2* | 2343-2373 | + | 1.12E-03 | ATTTAACTTAGGGCCAACCCGTCTCTGTGGC |
