# Supplementary material for: Genetic therapy in a mitochondrial disease model suggests a critical role for liver dysfunction in mortality
Source: eLife. 2022 Nov 21;11:e65488. doi: 10.7554/eLife.65488 (PMC9859037; doi:10.7554/eLife.65488)
Supplement: Transparent reporting form [file elife-65488-transrepform1.docx]

***eLife’s* transparent reporting form**

We encourage authors to provide detailed information *within their submission* to facilitate the interpretation and replication of experiments. Authors can upload supporting documentation to indicate the use of appropriate reporting guidelines for health-related research (see [EQUATOR Network](http://www.equator-network.org/%20)), life science research (see the [BioSharing Information Resource](https://biosharing.org/)), or the [ARRIVE guidelines](http://www.plosbiology.org/article/info:doi/10.1371/journal.pbio.1000412) for reporting work involving animal research. Where applicable, authors should refer to any relevant reporting standards documents in this form.

If you have any questions, please consult our Journal Policies and/or contact us: [editorial@elifesciences.org](mailto:editorial@elifesciences.org).

**Sample-size estimation**

- You should state whether an appropriate sample size was computed when the study was being designed
- You should state the statistical method of sample size computation and any required assumptions
- If no explicit power analysis was used, you should describe how you decided what sample (replicate) size (number) to use

Please outline where this information can be found within the submission (e.g., sections or figure legends), or explain why this information doesn’t apply to your submission:

No explicit power analysis and sample size of the experiments were based on prior published protocols. Sample sizes have been described in the materials and methods and in figure legends.

**Replicates**

- You should report how often each experiment was performed
- You should include a definition of biological versus technical replication
- The data obtained should be provided and sufficient information should be provided to indicate the number of independent biological and/or technical replicates
- If you encountered any outliers, you should describe how these were handled
- Criteria for exclusion/inclusion of data should be clearly stated
- High-throughput sequence data should be uploaded before submission, with a private link for reviewers provided (these are available from both GEO and ArrayExpress)

Please outline where this information can be found within the submission (e.g., sections or figure legends), or explain why this information doesn’t apply to your submission:

Replicate information for each experiment is described in Materials and methods.

Data inclusion criteria have been described in Materials and Methods.

Raw C_q_ values have been provided for each of the replicates for the experiment where expression of mitochondrial transcripts and *lrpprc* has been measured. Please see the source file information below for more details.
RNA sequencing FASTQ raw reads have been uploaded for each of the replicates on NCBI SRA repository ID PRJNA683704. (embargo till the date of publication of the study).

**Statistical reporting**

- Statistical analysis methods should be described and justified
- Raw data should be presented in figures whenever informative to do so (typically when N per group is less than 10)
- For each experiment, you should identify the statistical tests used, exact values of N, definitions of center, methods of multiple test correction, and dispersion and precision measures (e.g., mean, median, SD, SEM, confidence intervals; and, for the major substantive results, a measure of effect size (e.g., Pearson's r, Cohen's d)
- Report exact p-values wherever possible alongside the summary statistics and 95% confidence intervals. These should be reported for all key questions and not only when the p-value is less than 0.05.

Please outline where this information can be found within the submission (e.g., sections or figure legends), or explain why this information doesn’t apply to your submission:

Description and justification for statistical analysis methods have been described in the materials and methods and supplementary methods. Information is also provided in the figure legends with exact p-values.

We utilized violin plots and/or presented the data points shown to maximize the visualization of data and its distributions.

•Exact values of N and statistical tests utilized can be found in the figure legends and results section.

(For large datasets, or papers with a very large number of statistical tests, you may upload a single table file with tests, Ns, etc., with reference to sections in the manuscript.)

**Group allocation**

- Indicate how samples were allocated into experimental groups (in the case of clinical studies, please specify allocation to treatment method); if randomization was used, please also state if restricted randomization was applied
- Indicate if masking was used during group allocation, data collection and/or data analysis

Please outline where this information can be found within the submission (e.g., sections or figure legends), or explain why this information doesn’t apply to your submission:

The group allocation for all experiments is described in Materials and methods section.

**Additional data files (“source data”)**

- We encourage you to upload relevant additional data files, such as numerical data that are represented as a graph in a figure, or as a summary table
- Where provided, these should be in the most useful format, and they can be uploaded as “Source data” files linked to a main figure or table
- Include model definition files including the full list of parameters used
- Include code used for data analysis (e.g., R, MatLab)
- Avoid stating that data files are “available upon request”

Please indicate the figures or tables for which source data files have been provided:

**Figure 3- Source Data 1:**

Numeric data for the relative expression of *lrpprc* transcript in *lrpprc^+/+^* and homozygous mutant larvae, *lrpprc^-/-^*.

**Figure 4 - Source Data 1:**

A**.** Data for the survival percentage of *lrpprc^+/+^*, *lrpprc^GBT0235/+^* and *lrpprc^GBT0235/GBT0235^*.

B. Numeric data for the relative expression of mitochondrial encoded transcripts in *lrpprc^+/+^* and homozygous mutant larvae, *lrpprc^GBT0235/GBT0235^*.

C. Data for lactate measurements in *lrpprc^+/+^* and *lrpprc^GBT0235/GBT0235^*.

**Figure 5- Source Data 1:**

A. Numeric data for the birefringence measurements in *lrpprc^+/+^* and homozygous mutant larvae, *lrpprc^GBT0235/GBT0235^*.

Figure supplement 1: Numeric data for the birefringence measurements in 4 dpf *lrpprc^+/+^*, homozygous mutant larvae, *lrpprc^GBT0235/GBT0235^,* and liver-specific rescued larvae, *Tg(fabp10:Cre)lrpprc^GBT0235/GBT0235^*.

**Figure 7- Source Data 1:**

D. Numeric data for the oil red area in *lrpprc^+/+^ and* *Tg(fabp10:Cre)lrpprc^+/+^*, homozygous mutant larvae, *lrpprc^GBT0235/GBT0235,^* and liver-specific rescued homozygous mutant larvae, *Tg(fabp10:Cre)lrpprc^GBT0235/GBT0235^.*

E. Data for the survival percentage of *lrpprc^+/+^*, *lrpprc^GBT0235/GBT0235^* and larvae, *Tg(fabp10:Cre)lrpprc^GBT0235/GBT0235^.*

**Figure 8- Source Data 1:**

Data for the peak areas per larval equivalent and normalized to the TopFluor cholesterol peak across *lrpprc^+/+^*, *lrpprc^GBT0235/GBT0235,^* and larvae, *Tg(fabp10:Cre)lrpprc^GBT0235/GBT0235^.*

Supplementary file 1. Supplementary tables.(A) List of oligonucleotides used in the study. (B) Prediction of Human LRPPRC binding sites within the human mitochondrial genome (NC_012920.1). The top twenty matches among both strands of the complete mitochondrial genome are shown. The p-values were calculated with the FIMO program and were used for ranking. (C) Prediction of zebrafish Lrpprc binding sites within the zebrafish mitochondrial genome (NC_002333.2). The top twenty matches among both strands of the complete mitochondrial genome are shown. The p-values were calculated with the FIMO program and were used for ranking.

Supplementary file 2: Gene set enrichment analysis for *lrpprc* homozygous mutants.

Raw dataset for the RNA sequencing has been uploaded on NCBI SRA: ID PRJNA683704
